# A Panel of E2F Target Gene Signature Predicting the Prognosis of Hepatocellular Carcinoma

**DOI:** 10.3389/fgene.2022.879299

**Published:** 2022-05-03

**Authors:** Wenmin Hu, Yongmei Shi, Tongqin Han, Caiyun Liu, Xipeng Cao, Guangjun Shi, Wenjing Zhu

**Affiliations:** ^1^ School of Medicine and Pharmacy, Ocean University of China, Department of Pulmonary and Critical Care Medicine, Qingdao Municipal Hospital, Qingdao, China; ^2^ Department of Gynecology, Qingdao Municipal Hospital, School of Medicine, Qingdao University, Qingdao, China; ^3^ Department of General Practice, Qingdao Municipal Hospital, School of Medicine, Qingdao University, Qingdao, China; ^4^ Department of Hepatobiliary Surgery, Qingdao Municipal Hospital, Qingdao University, Qingdao, China; ^5^ Clinical Research Center, Qingdao Municipal Hospital, Qingdao University, Qingdao, China; ^6^ Respiratory Disease Key Laboratory of Qingdao, Qingdao Municipal Hospital, Qingdao University, Qingdao, China

**Keywords:** E2F target gene, risk, gene signature, prognosis, hepatocellular carcinoma

## Abstract

Hepatocellular carcinoma is one of the most malignant tumors, and the therapeutic effects of traditional treatments are poor. It is urgent to explore and identify new biomarkers and therapeutic targets to develop novel treatments which are individualized and effective. Three hallmarks, including E2F targets, G2M checkpoint and DNA repair, were collected by GSEA analysis. The panel of E2F-related gene signature consisted of five genes: HN1, KIF4A, CDCA3, CDCA8 and SSRP1. They had various mutation rates ranging from 0.8 to 5% in hepatocellular carcinoma, and patients with gene mutation had poorer prognosis. Among these genes, HN1 has the greatest mutation rate, and SSRP1 has the greatest impact on the model with a B (COX) value of 0.8842. Patients with higher expression of these genes had poorer prognosis. Kaplan-Meier curves in stratified survival analysis confirmed that patients with high risk scores had poor prognosis (*p* < 0.05). The results of univariate and multivariate COX survival analysis showed that risk score was closely related to the overall survival of patients with hepatocellular carcinoma. For clinical validation, we found that all the genes in the model were upregulated in hepatocellular carcinoma tissues compared to normal liver tissues, which was consistent with the previous results we obtained. Our study demonstrated that a panel of E2F target genes signature including five genes could predict the prognosis of hepatocellular carcinoma. This panel gene signature can facilitate the development of individualized and effective treatment for hepatocellular carcinoma.

## Introduction

Hepatocellular carcinoma (HCC) remains one of the most prevalent malignancies, which is the most ordinary form of primary carcinoma of the liver (Llovet et al., 2016). HCC has become the second leading cause of cancer death. It is hard to detect at the early stage, and more than 70% of HCC patients are diagnosed at an advanced stage, leading to dismal prognosis of patients with HCC (Kim and Park 2018). Although the advanced treatments such as surgical resection have emerged (Di Sandro et al., 2019), they are only suitable for 15% of the patients with HCC. The rest of the patients have to turn to other treatments due to their dismal general status, metastatic disease or imperfect liver function (e.g., underlying cirrhosis) (Chen et al., 2014; Chi et al., 2020), such as gene therapy (Kai Wang et al., 2021), immunotherapy (Aravalli and Steer 2017; Ringelhan et al., 2018), etc. The current advances in HCC treatment do not optimize the poor response of patients to treatment, and the long-term outlook of advanced stage patients with HCC remains bleak (Wang et al., 2019). The mechanism underlying HCC has not been thoroughly investigated. An improved understanding of its biology and Achilles heels might facilitate the development of novel, effective therapies for HCC.

E2F, which encodes a series of transcription factors, refers to a multifunctional transcription factor family. Due to their highly similar DNA binding domains, all the E2F-associated transcription factors can directly interact with the classical E2F consensus DNA binding sequence (TTCCCGCC) (Iaquinta and Lees 2007) which is extensively found in genes involved in mitosis, DNA synthesis, and cell cycle progression. Recent studies have revealed that the E2F family plays a crucial role in the regulation of tumor cell cycle (Kent and Leone 2019), DNA damage response (Segeren et al., 2020), cell differentiation and cell death (Chen et al., 2009) by linking with common DNA binding sequences, subsequently affecting tumor cell proliferation and invasion. A large body of evidence has demonstrated that E2Fs play important roles in tumorigenesis and cancer development by controlling their downstream target factors in a variety of cancers (Lee et al., 2015; Michaloglou et al., 2018; Yao et al., 2020).

Recently, with the advances in genome-sequencing technologies and bioinformatics, we get a better understanding of tumorigenesis and cancer development. Accumulating evidence (Li et al., 2019; Zheng et al., 2019; Tang et al., 2020) has showed that a variety of gene sets function as biomarkers in the vast majority of cancers, and changes in many marker genes associated with survival and prognosis have been revealed through data mining. Moreover, compared with single marker genes, multigene prognostic signatures can better predict the prognosis. Especially, polygenic prognostic features of messenger RNA (mRNA) have better prognostic accuracy compared with non-coding prognostic genes, which thereby provide more effective and personalized treatments (Karlsson and Larsson 2016; Han et al., 2020; Han et al., 2021). Identifying mRNA and hallmark gene sets has become a significant tool for clinical treatment for cancer. Despite the accumulating evidence regarding the prognostic roles of mRNA and hallmark gene sets in cancer, they were less studied in HCC. Thus, deep mining of publicly available genomic datasets and the discovery of more effective and potential prognostic mRNA biomarker gene sets for HCC are of paramount importance, providing the prognostic stratification and personalized treatment for HCC patients.

In this work, we screened out three gene sets with prognostic significance for HCC using gene set enrichment analysis (GSEA), in which the E2F target gene set consisted of 197 E2F target genes. After further analysis, a panel of gene signature containing five E2F target genes was established, with the aim of accurately predicting the prognosis of HCC patients. Our findings shed new insight on the gene sets which exerted significant effects on the prognosis of patients with HCC. Collectively, the results might facilitate the development of novel therapies for HCC patients.

## Materials and Methods

### Datasets

The mRNA expression profiles and the corresponding clinical information about HCC patients were obtained from the Cancer Genome Atlas (TCGA) database (including HCC and normal tissue specimens). The data are available for download in the GDC Data Portal: https://portal.gdc.cancer.gov/. The entire TCGA cohort (*N* = 368) was randomly divided into two groups, a test set (*N* = 245) and a validation set (*N* = 123). And the detailed clinicopathological parameters of HCC patients in each set, including age, gender, TNM stage, stage, histologic grade, tumor status and family history were illustrated in Table 1. Three datasets, GSE101685 (*N* = 32), GSE101728 (*N* = 14) and GSE14520 (*N* = 221) can be downloaded from the Gene Expression Omnibus database (GEO) (https://www.ncbi.nlm.nih.gov/geo/). And the HCC cohort of International Cancer Genome Consortium (ICGC) can also be downloaded (https://dcc.icgc.org/). The data from the TCGA, GEO and ICGC datasets are publicly available. Mutation data are derived from the cBioPortal for Cancer Genomics. 3 options, including “median”, “Auto select best cutoff” and “Trichotomization”, were existed on the Kaplan-Meier survival analysis website. In our study, the expression of each gene was divided into low- and high-groups according to the best cutoff (http://kmplot.com/analysis/index.php?p=service&cancer=liver_rnaseq).

**TABLE 1:** Clinical characteristics of patients with hepatocellular carcinoma in each set.

Clinical Characteristics	Test Set (%)	Validation Set (%)	Entire Set (%)
**Age**			
<60	54 (43.9)	119 (47.0)	173 (46.0)
≥60	69 (56.1)	134 (53.0)	203 (54.0)
**Gender**			
Male	85 (69.1)	170 (66.9)	255 (67.6)
Female	38 (30.9)	84 (33.1)	122 (32.4)
**TNM Stage**			
T1+T2	96 (80)	184 (72.4)	280 (74.9)
T3+T4	24 (20)	70 (27.6)	94 (25.1)
**Stage**			
Stage1+Stage2	87 (78.4)	175 (72.3)	262 (74.2)
Stage3+Stage4	24 (21.6)	67 (27.7)	91 (25.8)
**Grade**			
Grade1+Grade2	93 (78.2)	142 (56.1)	235 (63.2)
Grade3+Grade4	26 (21.8)	111 (43.9)	137 (36.8)
**Cancer Status**			
Tumor Free	79 (73.1)	157 (65.1)	236 (67.6)
With Tumor	29 (26.9)	84 (34.9)	113 (32.4)
**Family History of Hepatocellular Carcinoma**			
No	48 (57.1)	164 (67.8)	212 (65.0)
Yes	36 (42.9)	78 (32.2)	114 (35.0)

### Collection of the Tissue Specimens

In this study, the tumorous and matched non-neoplastic tissues were taken from 21 HCC patients undergoing surgical treatment at the Department of Thoracic Surgery at Qingdao Municipal Hospital, Qingdao University, between July 2020 and June 2021. This study was approved by the Medical Ethics Committee of Qingdao Municipal Hospital, Qingdao University (Qingdao, China). All enrolled patients signed the written informed consent form according to relevant regulations. The tissues of HCC patients and matched adjacent tumor controls were frozen immediately in liquid nitrogen after surgical resection and then stored at −80°C before use.

### Gene Set Enrichment Analyses

GSEA (Gene Set Enrichment Analyses) was performed to find the enriched gene sets affecting the prognosis of HCC patients. The hallmark gene sets screened out using GSEA were considered statistically significant with |NES|>1, NOM P-val<0.05 and FDR q-val<0.25.

### Establishment and Verification of the Prognostic Gene Signature

Univariate Cox regression analysis was performed to identify the prognostic genes with *p* < 0.001. Subsequently, using multivariate Cox regression analysis of hub genes, we established the prognostic gene signature. The unique risk value for each HCC patient was calculated according to the following risk score formula. Risk score = *β*1*expression of HN1 + *β*2*expression of KIF4A+ *β*3*expression of CDCA3 + *β*4*expression of CDCA8 + *β*5*expression of SSRP1. HCC patients were classified into a high-risk cohort (above the median) and a low-risk cohort (below the median) using the median risk score as the cutoff. The Kaplan-Meier method followed by the log-rank test was used to draw the survival curves based on the expression of five genes and overall survival (OS), progression-free survival (PFS), relapse-free survival (RFS), and disease-specific survival (DSS). The prediction model was validated using time-dependent receiver operating characteristic (ROC) curves together with Kaplan-Meier plot curves.

### Validating the Expression of the Five E2F Target Genes in Surgical Tissues by Quantitative Real-Time PCR (RT-qPCR)

To verify the accuracy of the established risk model, RT-qPCR assay was performed in the 21 tumor tissues and matched normal liver specimens, which detected the expression of five E2F target genes. Total RNA was extracted from patients with HCC using TRIgent^®^ reagent. The RNA concentration and the A260/A280 ratio were detected using ultramicro spectrophotometer (NanoVue, America), and the A260/A280 ratio in the range of 1.8–2.0 was regarded as acceptable. cDNAs were synthesized from total RNAs using *Evo M-MLV* RT Mix Kit with gDNA Clean for qPCR (Accurate Biology, China). Quantitative real-time polymerase chain reaction (RT-qPCR) was performed using the SYBR Green Premix Pro Taq HS qPCR Kit (Accurate Biology, China) in triplicate based on the CFX96™ Real-Time System (BIO-RAD, Singapore). Each PCR reaction system set up based on the manufacturer’s instructions was carried out with a sample volume of 10 μ L and amplified for 40 cycles. And the mRNA expression was normalized to GADPH. Relative RNA expression was calculated by the 2^−ΔΔCt^ method. Primer names and primer sequences were listed in following table (Shan et al., 2021).

**Table:** Primer Sequences of the Panel Genes

Primer Name	Primer Sequence
HN1 forward	GGCCTCTAATATCTTTGGGACA
HN1 reverse	TCACCTTCTCCCTTCAGATCTA
KIF4A forward	GCCGAGATAGAGACAGAGTTAC
KIF4A reverse	CACACTTCTCGCATTTTCTCAA
CDCA3 forward	TCTCCTACTCTTGGTATTGCAC
CDCA3 reverse	TACTTCACTCAGCTGTTTCACC
CDCA8 forward	CCGTGAAGTGGAAATACGAATC
CDCA8 reverse	GGATCTCGATGTTGTAGAGGTT
SSRP1 forward	GTCTGTTTTTGTTACCCCACAA
SSRP1 reverse	TTCTTCCTCGTTCATGTTCAGA
GAPDH forward	CAACGTGTCAGTGGTGGACCTG
GAPDH reverse	GTGTCGCTGTTGAAGTCAGAGGAG

### Statistical Analysis

All the statistical analyses were performed using SPSS software, version 24.0 (SPSS, Inc., Chicago, IL, United States) and GraphPad Prism 7.0 software (GraphPad, United States), and values were presented as mean ± S.D. or mean ± SEM of replicates. As for continuous variables fitting normal distribution between binary groups, student’s t-test (paired and unpaired) was used. Otherwise, we used Wilcoxon signed-rank test and Wilcoxon rank-sum test. Pearson χ^2^ test was performed to analyze the association between the expression of five genes and the pathological characteristics of HCC patients. In all the tests, a *p*-value less than 0.05 indicated statistical significance.

## Results

### Preliminary Screening of Hallmark Gene Sets by GSEA

**FIGURE 1:**
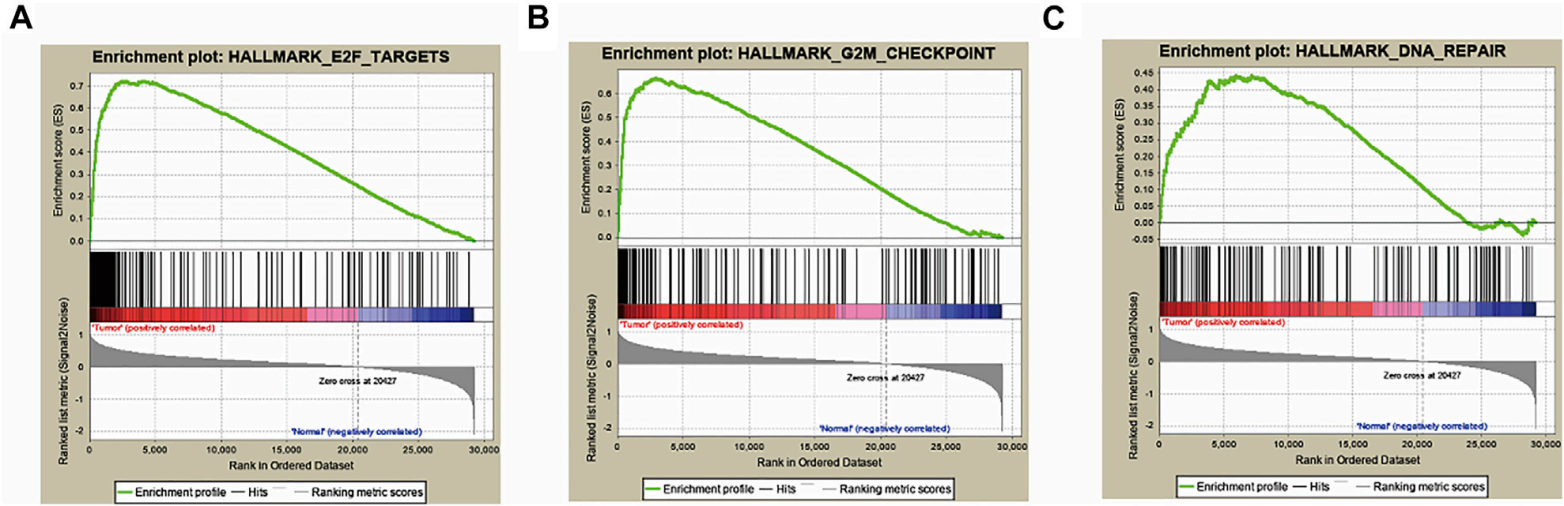
Three hallmarks, including E2F targets, G2M checkpoint and DNA repair, were collected by GSEA analysis **(A)** E2F target gene set. **(B)** G2M checkpoint gene set. **(C)** DNA repair gene set.

To explore the gene sets associated with poor prognosis of HCC, we included 422 HCC and adjacent normal tissues from TCGA database to detect the relationship between gene expression and overall survival via comparing their tumor tissues and the matched adjacent normal tissues. Thus, the expression data of 29,226 mRNAs were captured. Then, GSEA was used to screen out the hallmark gene sets, which were valuable for the prognosis of HCC patients, and three hallmark gene sets were screened out, including E2F targets, G2M checkpoint and DNA repair hallmark gene sets, with |NES|>1, NOM p-val<0.05 and FDR q-val<0.25, illustrated in Figures 1A–C and Table 2. Our research team has published our results showing that DNA repair gene set was correlated with HCC and an mRNA signature of seven mRNAs could be as a prognostic biomarker for HCC (Zhu et al., 2021). The E2F target gene set involved in a variety of biological processes was significantly associated with tumorigenesis and cancer development (Lee et al., 2015; Michaloglou et al., 2018; Yao et al., 2020). However, it must be noted that the roles of the E2F target gene set in HCC have not yet been studied. Therefore, we will investigate how the E2F target gene set affects HCC in the future.

**TABLE 2:** Hallmark gene sets were enriched in hepatocellular carcinoma.

GS Follow Link to MSigDB	Size	|NES|	NOM p-val	FDR Q-Val	Rank at MAX
E2F TARGETS	197	2.071552	0.001961	0.007023	4039
G2M CHECKPOINT	195	2.028344	0.005693	0.005554	2901
DNA REPAIR	141	1.649191	0.033333	0.128221	7117

A total of 197 genes were screened out from the E2F target gene set, as determined by GSEA. Then we conducted a univariate Cox regression to further screen out prognostic genes with normalized *p* < 0.001 from the 197 genes, yielding 32 genes that were independently associated with the OS of HCC patients. After primary filtration, those mRNAs were further investigated for their correlations with HCC progression and for their prognostic values. After a stepwise multivariate Cox regression for these genes, five genes, including HN1, KIF4A, CDCA3, CDCA8, and SSRP1, turned out to be independently correlated with HCC patients’ prognosis. Among these five genes, CDCA8 demonstrated the strongest association with prognostic outcomes in HCC patients, and SSRP1 had the greatest weight coefficient (Table 3). And SSRP1 has been reported as a basic transcription factor for most genes (Zhang et al., 2015).

**TABLE 3:** The detailed results from multivariate COX survival analysis of the selected prognostic mRNAs.

Gene	Ensemble ID	HR	B(COX)	*p* Value
HN1	ENSG00000189159	1.383224	0.3915	3.85E-05
KIF4A	ENSG00000090889	1.265077	−0.3864	7.36E-05
CDCA3	ENSG00000111665	1.303445	−0.2886	9.37E-05
CDCA8	ENSG00000134690	1.482907	0.4415	1.15E-07
SSRP1	ENSG00000149136	2.246367	0.8842	1.54E-05

### Establishment of a Prognostic Gene Signature of Five Genes

**FIGURE 2:**
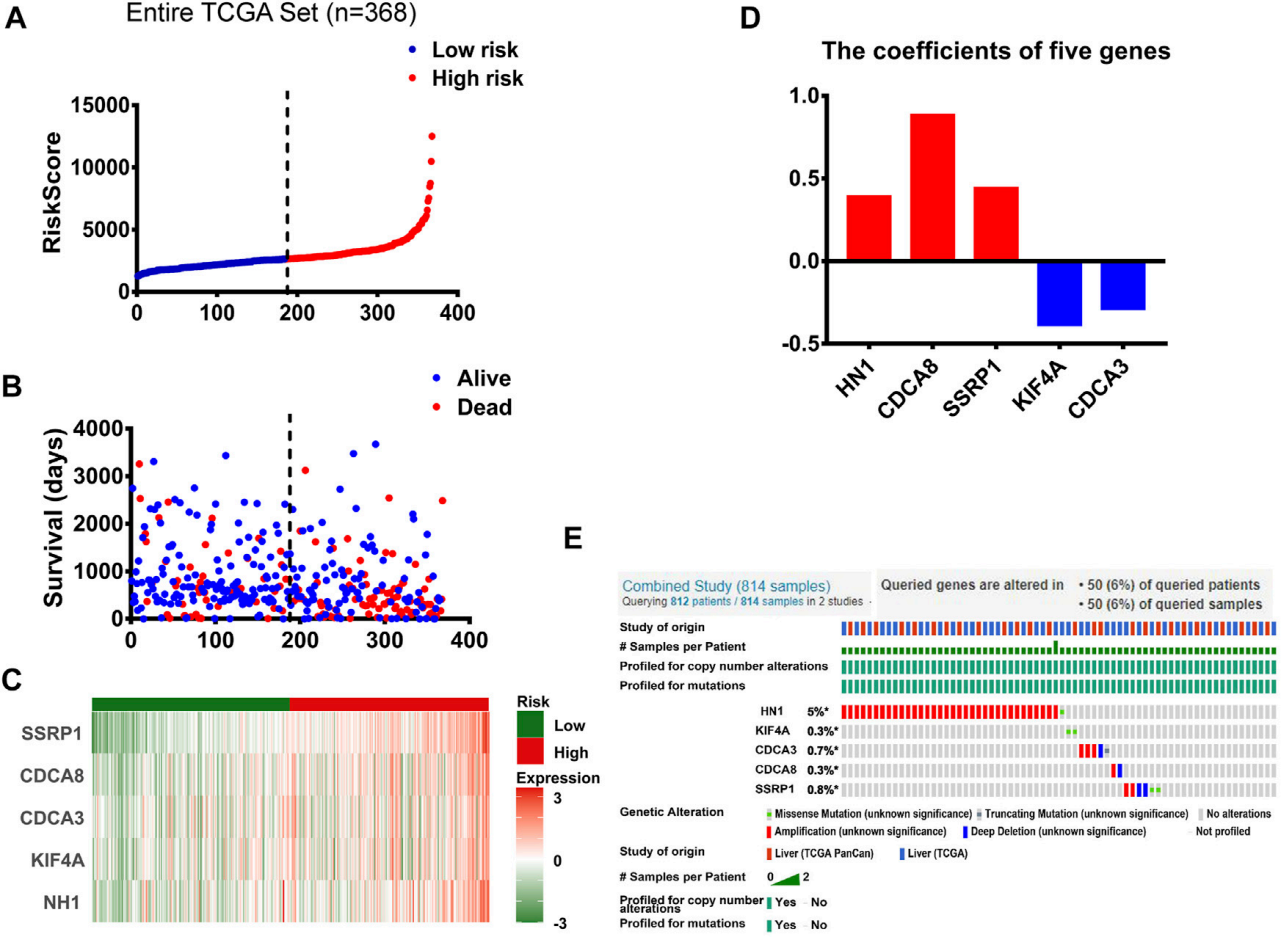
The panel of E2F target genes signature **(A)** The distribution of risk scores in patients with HCC. **(B)** The survival time and survival status of patients with HCC ranked by risk scores **(C)** The distribution of expression of the five genes in heatmap in patients with HCC ranked by risk scores. **(D)** The coefficients of five genes. **(E)** Genetic alterations of five E2F target genes from TCGA PanCan and TCGA in patients with hepatocellular carcinoma.

We established a prognostic signature of five genes. The predictive signature was characterized by a linear combination of the expression levels of the identified five genes weighted by their relative coefficients in the multivariate Cox regression analysis. Risk score = 0.3915* expression of gene HN1 - 0.3864* expression of gene KIF4A- 0.2886 * expression of gene CDCA3 + 0.4415* Expression of gene CDCA8 + 0.8842 * expression of gene SSRP1 (Table 3 and Figure 2D). In Figure 2D, the prognosis of HCC patients was positively associated with three mRNA genes (HN1, CDCA8, and SSRP1), and negatively associated with the other two mRNAs (KIF4A and CDCA3). The risk score could be calculated by the multiplication of the regression coefficient of each selected mRNA and the normalized expression level of each patient. And the gene signature endowed a risk score for each patient. A greater regression coefficient indicates higher risk and a worse prognosis of HCC patients. Additionally, all the coefficients for HN1, CDCA8 and SSRP1 were positive, suggesting that elevated expression of the three genes were significantly correlated with shortened overall survival.

We ranked these patients based on their risk scores. Then, 368 cases with HCC downloaded from TCGA database were divided into low- and high-risk cohorts with the median risk score as the threshold value (Figure 2A). The analysis of the overall survival of HCC patients with the five-gene expression signature demonstrated that the high-risk group had significantly shorter overall survival and a significantly greater number of deaths than the low-risk group, as illustrated in Figure 2B and Supplementary Figure S1A. Furthermore, the heatmap clustering analysis of expression profiles for 5 genes showed that these genes were gradually upregulated as the risk scores increased in HCC patients (Figure 2C).

Moreover, based on Kaplan-Meier plot online analysis, the low and high groups were divided according to the best cutoff of each gene, which were on the Kaplan Meier online analysis website. And we probed not only the relationship between expression of five genes and OS, but also the associations of expression of five genes with progression-free survival (PFS), relapse-free survival (RFS), and disease-specific survival (DSS). Among them, the preferred efficacy endpoint for tumors was OS in clinical trials; the time from the start of grouping to the first progression of disease or death from any cause was referred to as PFS, which can better reflect tumor progression and predict clinical benefits; RFS, an important index of the efficacy evaluation, was constantly used in advanced tumors. The time of death from causes other than the study disease was not included in the DSS, which was also an important indicator for the evaluation of the efficacy in advanced tumors. The prognostic results showed that elevated expression of 5 genes were significantly correlated with declines in OS, PFS, RFS and DSS (Supplementary Figures S2, S3).

### Aberrant Expression of the Five-Gene Signature in Hepatocellular Carcinoma

**FIGURE 3:**
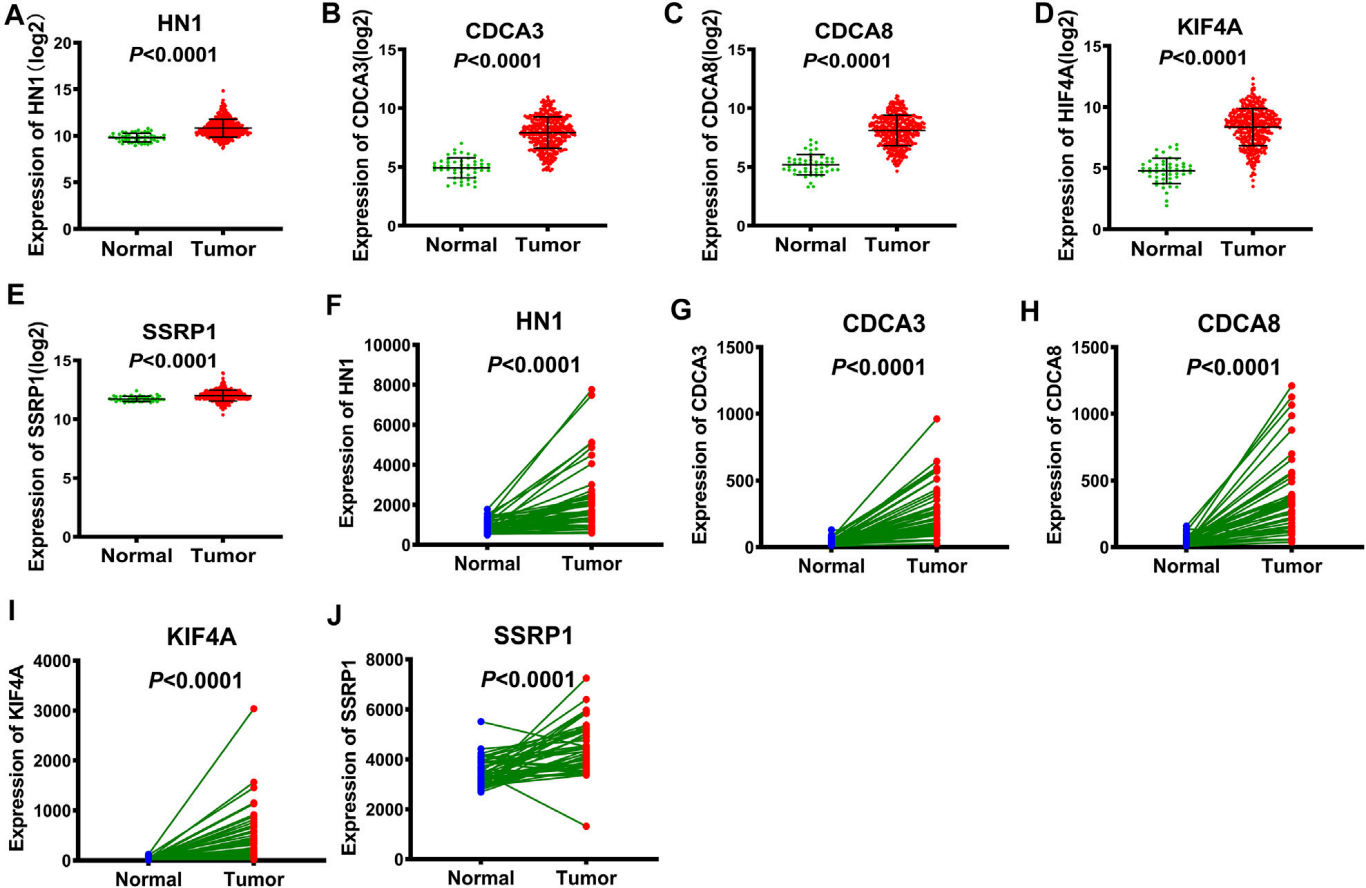
The expression of E2F target genes in tumor tissues versus normal tissues from TCGA **(A-E)** Wilcoxon rank-sum test, the expression of HN1, CDCA3, CDCA8, KIF4A and SSRP1 in tumor tissues versus normal tissues, respectively. **(F-J)** Wilcoxon signed-rank test, the expression of HN1, CDCA3, CDCA8, KIF4A and SSRP1 in tumor tissues versus normal tissues, respectively.

Using TCGA database, we obtained the expression data of these 5 genes in tumor tissues versus normal tissues. The expression levels of the five identified genes, in HCC tissues, were higher compared to those detected in the whole controls (*p* < 0.0001, Figures 3A–E). We further substantiated the associations between the expression of the five genes and the prognosis of HCC patients using gene expression data from 50 pairs of TCGA specimens analyzed by the Wilcoxon signed-rank test. Consistent with the above results, the Wilcoxon signed-rank test showed that the expression levels in HCC tumor tissues were significantly higher than those in their normal counterparts (*p* < 0.0001, Figures 3F–J).

To further confirm the expression patterns and the accuracy of the risk model of the five genes in the GEO database of HCC patients, these five genes were selected from two datasets, GSE101685 and GSE101728. The GSE101685 referred to the unpaired tumor tissues and adjacent normal tissues. There were statistical differences in the expression levels of the five genes between the unpaired tumor and normal tissues, suggesting higher expression of these 5 genes in HCC tissues (Supplementary Figures S4A–E). The GSE101728 referred to the paired tumor tissues and their adjacent normal tissues. And the GSE101728 suggested that these 5 genes were highly expressed in HCC tumor tissues compared with their corresponding noncancerous tissues, which was consistent with the result from the GSE101685.

We then used the cBioportal database to determine the types and frequency of the alterations of the five genes in HCC patients based on DNA sequencing data. Among them, HN1, also called HN1A, ARM2, or JPT1, which was a microtubule-associated protein located on human chromosome 17q25.2 (Sears 2004), had the highest genetic alteration frequency (Figure 2E). Current evidence has shown that HN1 can regulate the cell cycle, growth, and repair of the retinal cells and embryonic cells (Goto et al., 2012). In addition, HN1 has been reported to be of great significance for the prognosis of HCC patients (Liu et al., 2020; Pu et al., 2020).

Besides, HN1 had amplification and missense mutation, with the highest genetic alteration frequency of 5%. KIF4A had only missense mutation. And CDCA3 had three types of genetic alterations, namely amplification, deep deletion, and truncating mutation. Similarly, SSRP1 had three types of genetic alterations, including amplification, deep deletion, and missense mutation, with the second highest genetic alteration frequency of 0.8%. Although CDCA8 shared the same genetic alteration frequency with KIF4A, CDCA8 had two types of genetic alterations, i.e. amplification and deep deletion. (Figure 2E).

**FIGURE 4:**
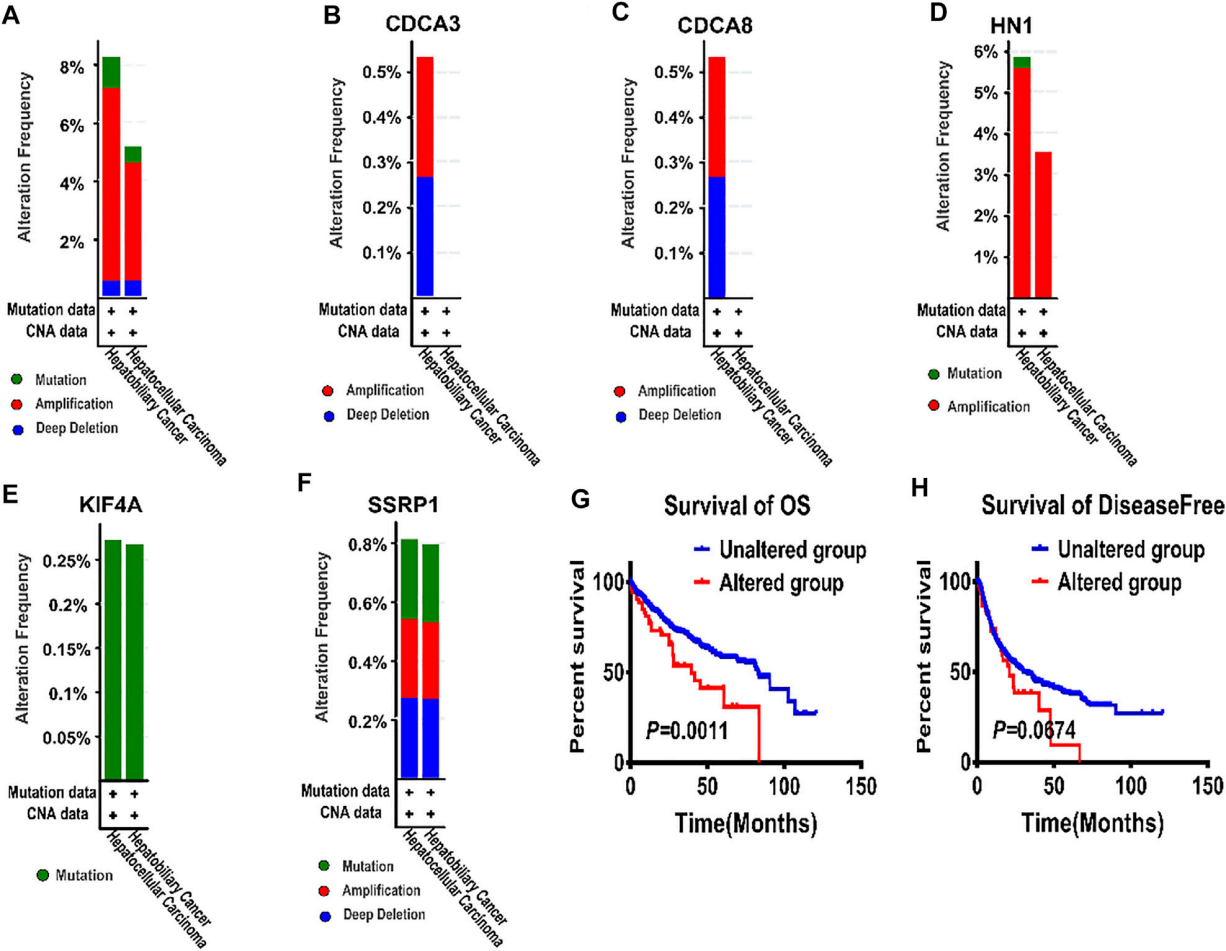
The alterations of E2F target genes in tumor tissues from cBioportal database **(A)** All alteration types across five genes in patients with hepatocellular carcinoma. **(B–F)** Genetic alterations of CDCA3, CDCA8, HN1, KIF4A, and SSRP1 were respectively described in **(B–F)** with specific alteration frequencies **(G)** The overall survival of hepatocellular carcinoma patients in unaltered and altered groups. **(H)** The disease-free survival of hepatocellular carcinoma patients in unaltered and altered groups.

Subsequently, the genetic alterations of these five genes were vividly described in Figures 4A–F. Firstly, there were three types of genetic alterations across the 5 genes, including mutation, amplification and deep deletion (as shown in Figure 4A). Secondly, the genetic alterations of the five genes were separately analyzed (as shown in Figures 4B–F). Finally, survival curves were used to analyze the associations between the five genes and the prognosis of HCC patients, showing that the overall survival (OS) and disease-free period of HCC patients were shortened after genetic alterations (Figures 4G,H), that is, the prognosis of HCC patients was directly affected by the alterations of the 5 target genes.

All the above results showed the abnormal expression of the five genes in patients with HCC. Consequently, the five target genes were of great significance to the prognosis of HCC patients.

### Risk Score, an Independent Prognostic Factor for Hepatocellular Carcinoma

**FIGURE 5:**
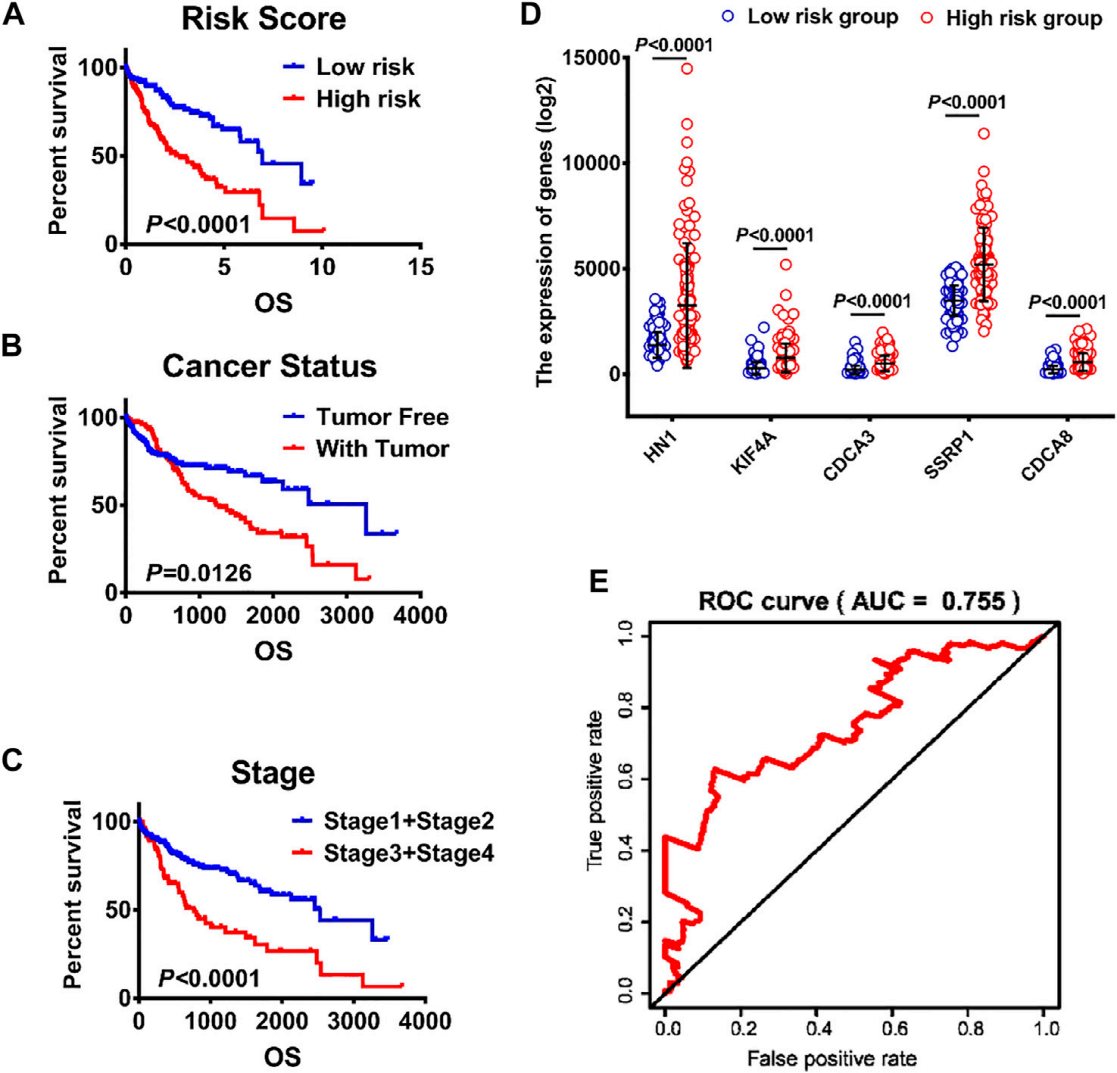
Analysis of clinicopathological parameters affecting the prognosis of hepatocellular carcinoma patients **(A)** K–M survival curves of patients in high-risk and low-risk groups. **(B–C)** The effects of different clinicopathological parameters including cancer and stage on patients’ Kaplan–Meier survival curves **(D)** The expression levels of HN1, KIF4A, CDCA3, SSRP1 and CDCA8 in low- and high-risk groups. **(E)** A ROC curve of patients with HCC from TCGA.

Univariate and multivariate Cox regression analyses were performed to determine whether the predictive value of the risk model was independent of traditional clinicopathological parameters. Univariate analysis showed that risk score (*p* < 0.05, HR = 1.708), family cancer history (*p* < 0.05, HR = 2.032), new event time (*p* < 0.05, HR = 0.998), T stage (*p* < 0.05, HR = 2.524), stage (*p* < 0.05, HR = 2.432) and cancer status (*p* < 0.05, HR = 2.836) were significantly correlated with the OS of HCC patients. The mortality score of HCC patients at high risk was 1.708 times greater than that of the patients at low risk (Table 4). Similar results were obtained when multivariate Cox regression analysis was further employed, confirming the independent prognostic roles of risk score, family cancer history, cancer status and new event time in overall survival time after adjusting for confounders including family cancer history, age, gender, new event time, tumor topography, stage and cancer status. Thus, greater risk scores were significantly associated with smaller OS in HCC patients (HR, 1.708; 95% CI, 1.122–2.602; *p* = 0.012), indicating that risk score can be used as an independent prognostic factor for HCC patients. Next, the results in the Cox regression analysis were verified by Kaplan-Meier curves followed by log-rank test, which revealed that poor prognosis of HCC patients had significant associations with high-risk score, presence of tumor, late clinical stage (Figures 5A–C).

**TABLE 4:** Univariate and multivariate analyses for predictors of overall survival of hepatocellular carcinoma patients in TCGA.

Factor	Univariate Analysis			Multivariate Analysis		
	HR	95%CI of HR	*p* Value	HR	95%CI of HR	*p* Value
**Risk Score**	1.708	1.122–2.602	0.012	0.526	0.312–0.885	0.016
Family Cancer History (Yes/No)	2.032	1.313–3.144	<0.0001	0.569	0.341–0.947	0.023
**Age**	1.706	1.111–2.619	0.013	0.717	0.423–1.214	0.216
Gender (Male/Female)	0.722	0.473–1.103	0.130	1.292	0.774–2.157	0.327
**New Event Time**	0.998	0.997–0.998	<0.0001	0.998	0.997–0.998	<0.0001
Tumor Topography (T1+T2/T3+T4)	2.524	1.774–3.591	<0.0001	1.251	0.162–9.635	0.83
Stage (Stage1+2/Stage3+4)	2.432	1.678–3.525	<0.0001	0.806	0.105–6.168	0.835
Cancer Status (Tumor Free/With Tumor)	2.836	1.787–4.500	<0.0001	0.266	0.112–0.630	0.003

### Verifying the Risk Scores of the mRNA Signature

The validity of the risk scores of the five mRNA was verified based on the time-dependent ROC curve and Kaplan-Meier survival curve methods. HCC patients were divided into high-risk and low-risk groups based on the median risk score. The forecasting capacity of the five-gene signature was assessed by calculating the AUC. As shown in Figure 5E, the AUC value was 0.755, indicating that risk score had high sensitivity and specificity in predicting the prognosis of HCC patients. In addition, as shown in Figure 5D, the gene expression levels of the high-risk cohort were dramatically higher compared to the low-risk cohort with statistical significance (*p* < 0.0001).

**FIGURE 6:**
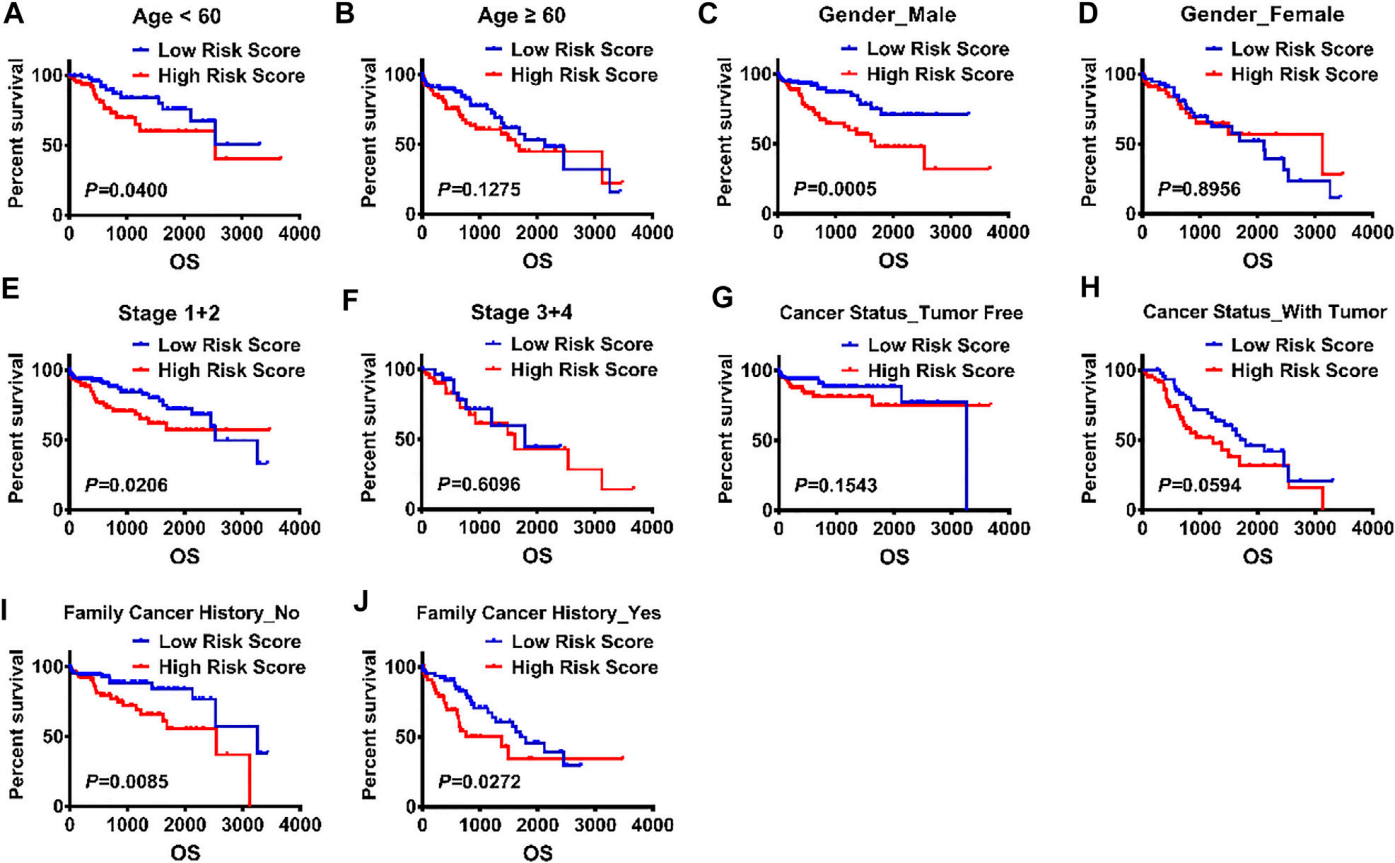
Validation of the risk scores for a panel of E2F target gene signature by KM plot. **(A-J)** KM survival analysis was used to explore the impact of risk score on the prognosis of HCC patients stratified by the clinical and pathological parameters, including age, gender, stage, cancer status, family cancer history, and risk score.

Further, Kaplan–Meier survival curves were used to analyze the survival and prognosis of HCC patients in high versus low risk groups, showing that the prognosis of low-risk group was much better than that of high-risk group (*p* < 0.0001, Figure 5A). Thus, we conducted stratified survival analysis to test the accuracy of risk score as a predictor for HCC in subgroups with different clinicopathological characteristics. The stratified survival analysis showed that the association between risk score and OS of HCC patients was more significant in patients aged <60 (*p* = 0.0400), males (*p* = 0.0005), stage I/II patients (*p* = 0.0206), those without tumor (*p* = 0.01543) and those without a family history of cancer (*p* = 0.0085) than those aged ≥60 (*p* = 0.1275), females (*p* = 0.8956), stage III/IV patients (*p* = 0.6096), those with tumor (*p* = 0.0594) and those with a family history of cancer (*p* = 0.0272) (Figures 6A–J).

**FIGURE 7:**
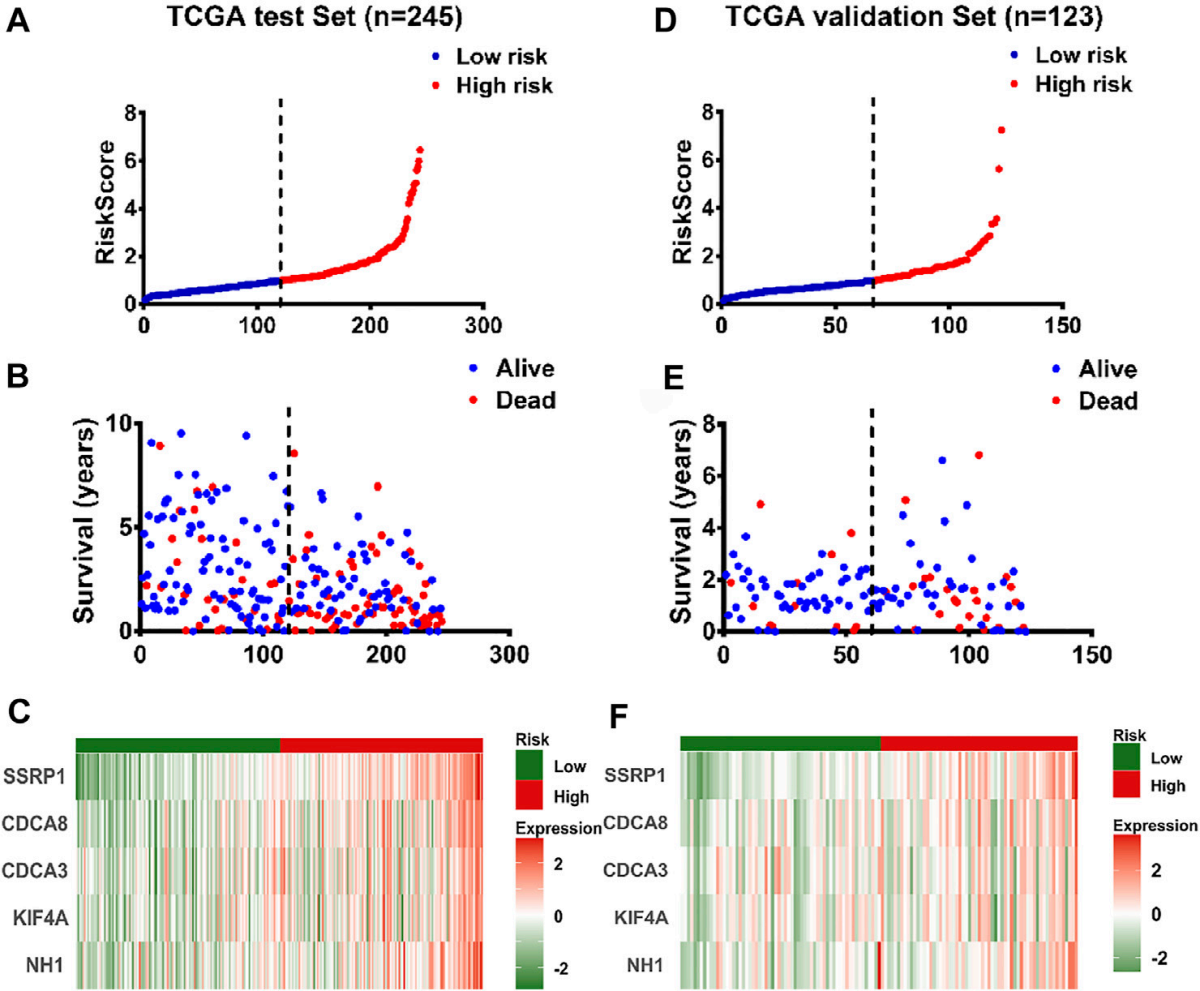
Validation of the panel of E2F target genes signature by test set and validation set **(A)** The distribution of risk scores in patients with liver cancer by TCGA test set (n = 245) **(B)** The survival time and survival status of patients with liver cancer ranked by risk scores by TCGA test set (n = 245) **(C)** The distribution of expression of the five genes in heatmap in patients with liver cancer ranked by risk scores by TCGA test set (n = 245) **(D)** The distribution of risk scores in patients with liver cancer by TCGA validation set (n = 123) **(E)** The survival time and survival status of patients with liver cancer ranked by risk scores by TCGA validation set (n = 123) **(F)** The distribution of expression of the five genes in heatmap ranked by risk score in patients with liver cancer by TCGA validation set (n = 123).

In addition, 368 HCC cases were obtained from TCGA database in our predictive signature, of whom 245 belonged to the test cohort and 123 belonged to the validation cohort. The two cohorts of patients were assigned to the low- and high-risk groups according to a cut-off median risk score (Figures 7A–F and Supplementary Figures S1B–C). Figures 7B,E and Supplementary Figures S1B–C showed the survival status of HCC patients in the test cohort and validation cohort, and the heatmap demonstrated that the expression levels of the five genes increased as the risk scores increased in HCC patients, in both the test group and validation group (Figures 7C,F). In addition, the validation group was used to test the accuracy of the gene signature.

The external validation HCC cohort from ICGC database and GSE14520 set were adopted to verify those results derived from TCGA database. There exist significant difference in the expression of five genes between cancer and normal tissues (Supplementary Figures S5A, D). The risk score of patients with advanced HCC was significantly higher than that of patients with early HCC (Supplementary Figures S5B). 228 HCC patients from ICGC database were divided into the high-risk (*n* = 114) and low-risk (*n* = 114) groups according to the median value of the risk score, as same the GSE14520 data set from GEO database. The results from ICGC database and GSE14520 data set showed that patients with high-risk score had a significantly greater number of deaths than the patients with low-risk score, and the expression of 5 genes was gradually upregulated along with the risk scores increased (Supplementary Figures S4E–H). In addition, the area of ROC curves for 1-year, 2-yesr, 3-years, and 5-years overall survival in ICGC cohort were 0.638, 0.673, 0.725 and 0.703, respectively (Supplementary Figure S5C), and these results showed that the prognostic model we constructed has good accuracy and specificity. All of the results from the ICGC and GEO database were in accord with those results derived from TCGA database.

**FIGURE 8:**
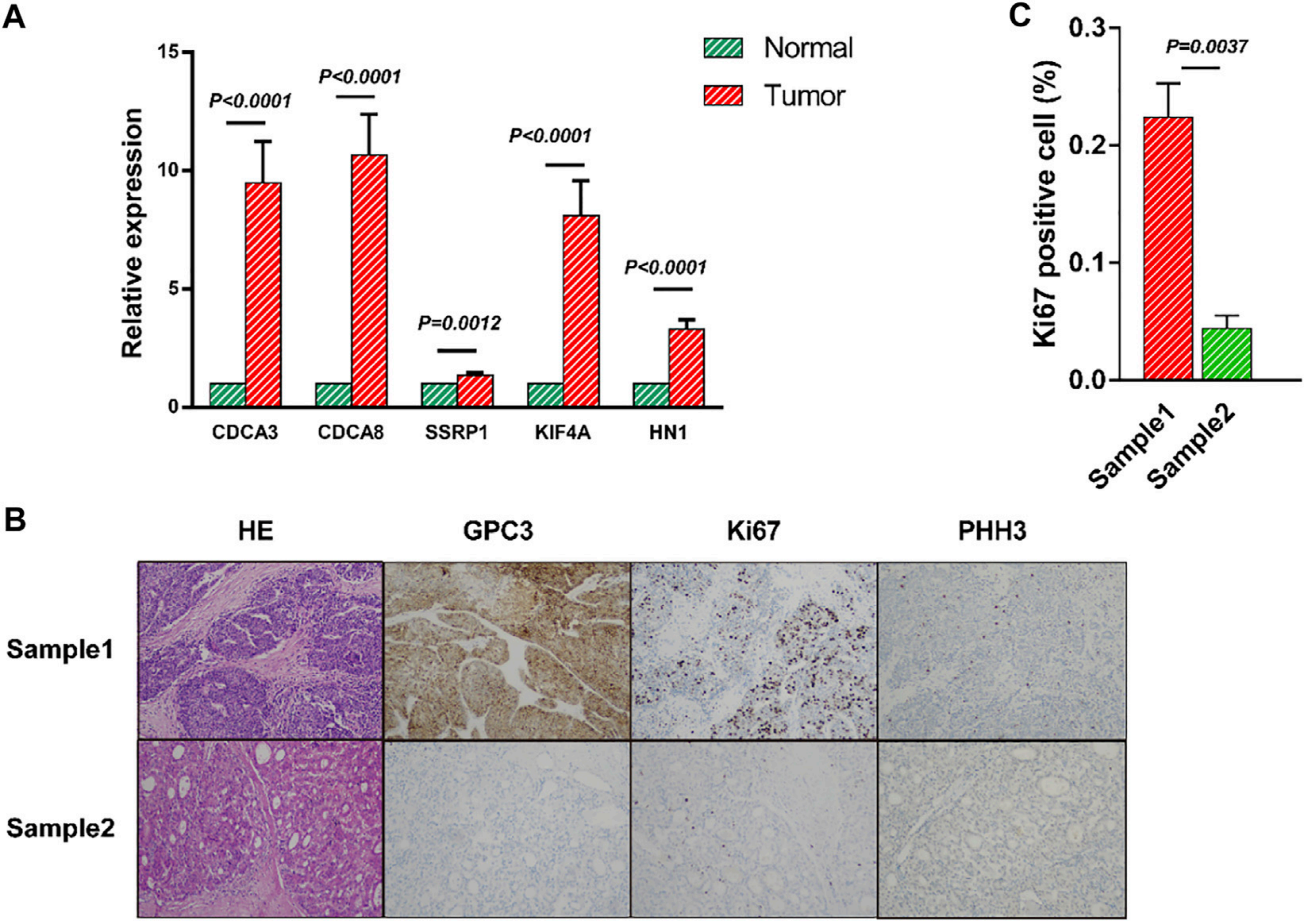
Validation of the mRNA panel signature in HCC patients from clinical tissue specimens **(A)** The mRNA relative expression of CDCA3, CDCA8, SSRP1, KIF4A and HN1 in HCC tissues was evaluated by qRT-PCR. **(B)** Representative hematoxylin-eosin (H&E) and immunohistochemistry (IHC) staining of GPC3, Ki67 and PHH3 in HCC patients. **(C)** There was significant difference between the Sample1 and Sample2 by Ki67 expression. All data are shown as the mean ± SEM.

To further confirm the above conclusions, the mRNA levels of the five genes were measured in 21 HCC tissues and their adjacent normal tissues by RT-qPCR. RT-qPCR showed aberrant expression of CDCA3, CDCA8, SSRP1, KIF4A and HN1, indicating that five genes were markedly upregulated in HCC tissues compared with adjacent normal tissues, which was in line with the prediction of our model (Figure 8A). Moreover, we calculated the risk score of each clinical patient according to the risk score formula, the results of hematoxylin-eosin (H&E) and immunohistochemical staining revealed that the patients with high-risk score had worse differentiation and higher clinical stages than the patients with low-risk score (Figures 8B,C and Supplementary Figures S6A–D).

Taken together, these results confirmed the accuracy of the panel gene signature and strongly proved that the risk score was a stable and accurate prognostic indicator for HCC.

## Discussion

Hepatocellular carcinoma (HCC), which is the leading cause of cancer-related death worldwide, is a molecularly heterogeneous disease. And the overall 5-years survival rate for HCC patients remains less than 20% (Nault and Villanueva 2021), thereby seriously threatening human health. Early diagnosis and application of novel therapy approaches including transplantation (Wang et al., 2018), genetic therapies, targeted therapies and immunotherapy (Rizvi et al., 2021) play critical roles in the prognosis of HCC patients. Nowadays, mRNA effects shown in genomic studies have received considerable attention. Many potentially valuable mRNAs have been identified to improve the clinical outcomes in HCC patients. However, the mortality rate of HCC patients has not appreciably improved, possibly due to deficiencies in the effective screening for molecular biomarkers. Therefore, there is an urgent need to find the molecular biomarkers which can predict the prognosis of HCC patients.

Presently, accumulating evidence indicates that the combination of messenger RNAs (mRNA) is more accurate in prognosis prediction compared with the non-coding genes (Li et al., 2020; Zhou et al., 2020). Therefore, establishing a gene set of mRNAs is an important approach to precise targeted therapy for HCC. However, studies reporting multiple mRNAs associated with the prognosis of HCC patients are scarce.

Our study aimed to investigate the prognostic role of mRNAs and construct a five-gene signature for predicting the prognosis of HCC patients. We analyzed the differences in mRNA expression between tumor tissues and normal tissues in HCC patients, and identified 3 significant marker gene sets with abnormal expression using GSEA, namely E2F target, G2M checkpoint and DNA repair marker gene sets. As previously described, E2F has become one of major transcriptional regulators of cell cycle dependent gene expression and takes part in many physiological and pathological processes, including tumor cell cycle (Kent and Leone 2019), DNA damage response (Segeren et al., 2020), cell proliferation, cell differentiation as well as cell death (Chen et al., 2009).

The univariate and multivariate Cox analyses of the E2F target gene set were conducted and we finally found five genes to be significantly correlated with the prognosis of HCC patients, including HN1, KIF4A, CDCA3, CDCA8 and SSRP1, which is consistent with many previous studies. HN1 was upregulated in liver cancer, which may promote tumor proliferation and invasion, accelerate tumor progression and cause poor prognosis of liver cancer (Liu et al., 2020). Overexpressed KIF4A facilitate cell proliferation, colony formation, and growth rate of HCC cells (Hu et al., 2019). Elevated expression of CDCA3 may affect the carcinogenic process of HCC by influencing the cell cycles, which was associated with poor OS, RFS, PFS and DSS (Zhihuai Wang et al., 2021). Advanced HCC patients had higher CDCA8 expression than patients with early HCC. Overexpressed CDCA8 and SSRP1 can be the potential predictors of the prognosis of HCC patients (Luo et al., 2021; Shuai et al., 2021). To our knowledge, our study is the first to focus on the combination of these five genes and will provide clinical implications for the development of new prognostic factors for HCC.

Accumulating evidence has revealed that multi-gene panel is more accurate in predicting the prognosis of HCC patients, compared with single mRNA genes (Yan et al., 2019; Zarębska et al., 2021). Therefore, a five-gene signature was constructed to predict the prognosis of HCC patients by GSEA, as well as univariate and multivariate Cox regression analyses. Then we partitioned patients into two groups according to the median risk score. We found that the expression of the 5 genes increased as the risk scores increased, and patients at high risk had shorter OS and more deaths, compared with the low-risk group (Figures 4A,F). The results revealed that the five genes showed significantly higher expression in tumor tissues compared to normal tissues from TCGA and GEO databases. Additionally, Kaplan-Meier plot online analysis showed that patients with high expression of the five genes had shorter OS, PFS, RFS as well as DSS than those with low expression.

Furthermore, the time-dependent ROC curve showed that the sensitivity and specificity of the prognostic model were great. And stratified analysis by different pathological parameters, including age, sex, tumor stage, tumor state and family tumor history, showed that the high-risk group had shorter OS and the risk score could be affected by these pathological parameters. Consistent with the results of the overall analysis, stratified analysis verified the reliability of the risk score model in predicting the prognosis of HCC patients.

However, there are several limitations in the present study. To our knowledge, TCGA and GEO databases are important tools for analysis of complex biomarkers, since they provided abundant gene sequencing data and clinical information available for analysis. Some candidate genes intervening prognosis might have been removed before establishing the prognostic model, which could decrease the performance of the risk model. Additionally, with the continuous progress of sequencing methods, more new genes are found, and more and more genes will be added to E2F target set. In the future, we will always pay attention to the research of E2F target set.

## Conclusion

Our results indicated that a panel of E2F target gene signature, comprising of HN1, KIF4A, CDCA3, CDCA8 and SSRP1, is a reliable tool for predicting the overall survival of Hepatocellular carcinoma patients, and provided significant reference of clinical risk for Hepatocellular carcinoma patients.

## Data Availability

The datasets presented in this study can be found in online repositories. The names of the repository/repositories and accession number(s) can be found below: https://portal.gdc.cancer.gov/https://www.ncbi.nlm.nih.gov/geo.
