# Supplementary material for: A Panel of E2F Target Gene Signature Predicting the Prognosis of Hepatocellular Carcinoma
Source: Front Genet. 2022 May 3;13:879299. doi: 10.3389/fgene.2022.879299 (PMC9110819; doi:10.3389/fgene.2022.879299)
Supplement: Supplementary file 1 [file DataSheet1.docx]

Supplementary Material

# Supplementary Figures

**
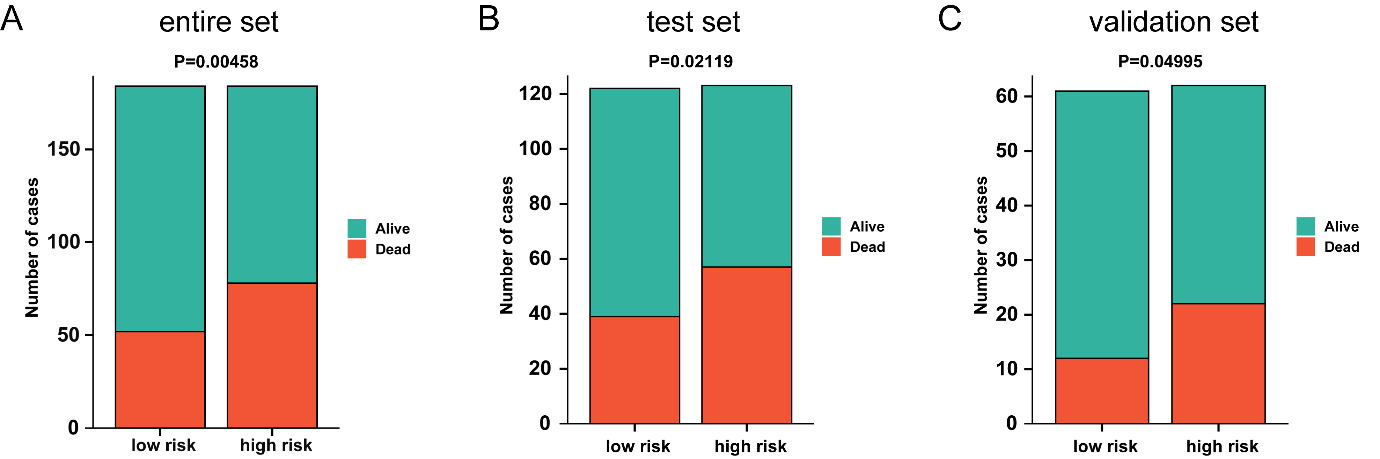
**

**Supplementary Figure 1 |** The boxplot of three sets. **(A)** The distribution of survival status in entire set of patients with HCC. **(B)** The distribution of survival status in test set of patients with HCC. **(C)** The distribution of survival status in validation set of patients with HCC.


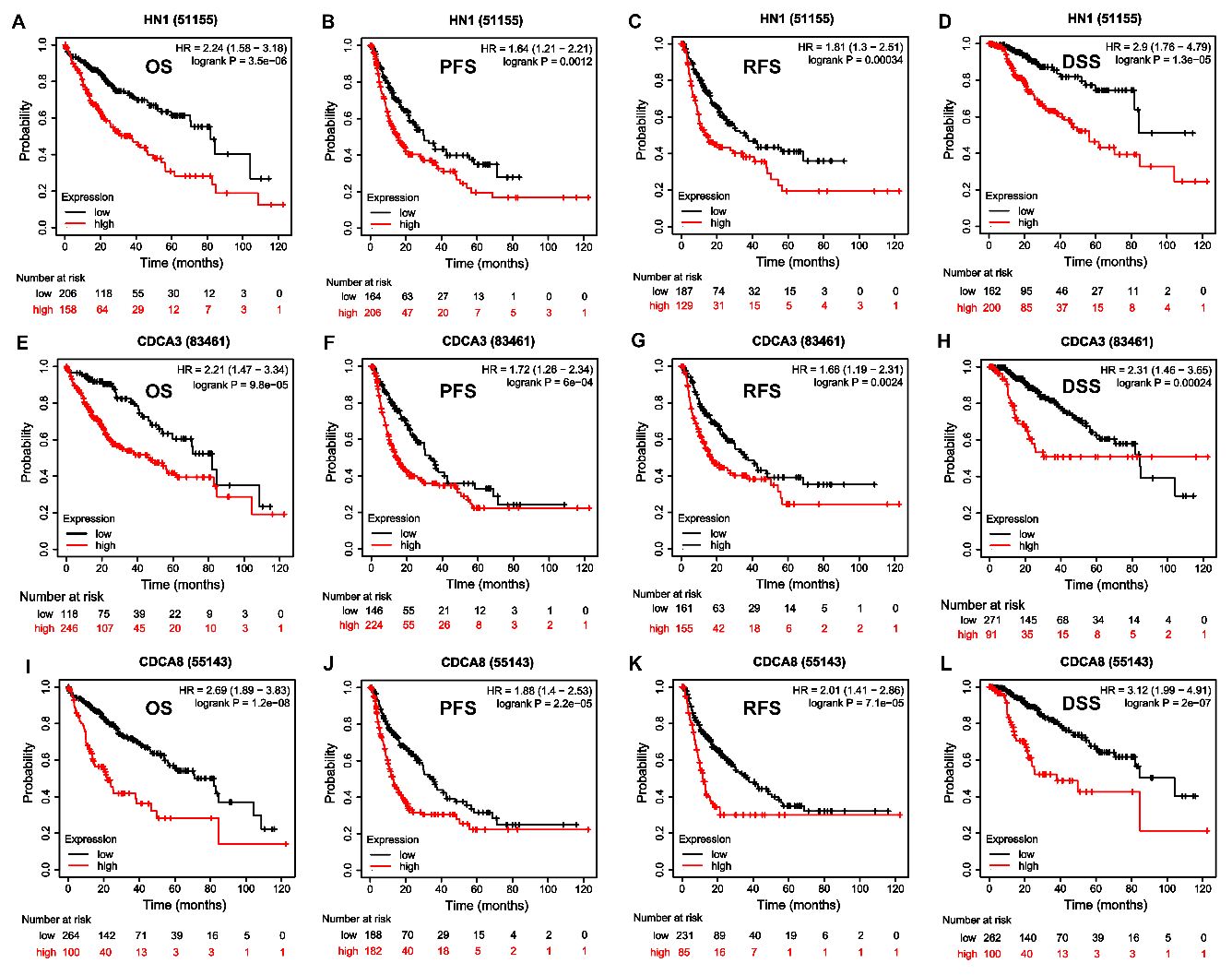


**Supplementary Figure 2 |** The effect of E2F target genes on prognosis of hepatocellular carcinoma patients**.** **(A-D)** The OS、PFS、RFS and DSS survival curves of HNI. **(E-H)** The OS、PFS、RFS and DSS survival curves of CDCA3. **(I-L)** The OS、PFS、RFS and DSS survival curves of CDCA8.


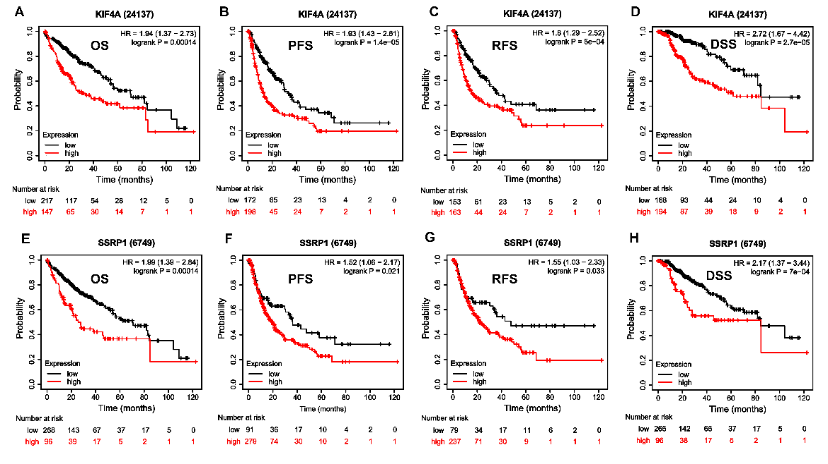


**Supplementary Figure 3 |** The effect of E2F target genes on prognosis of hepatocellular carcinoma patients. **(A-D)** The OS、PFS、RFS and DSS survival curves of KIF4A. **(E-H)** The OS、PFS、RFS and DSS survival curves of SSRP1.


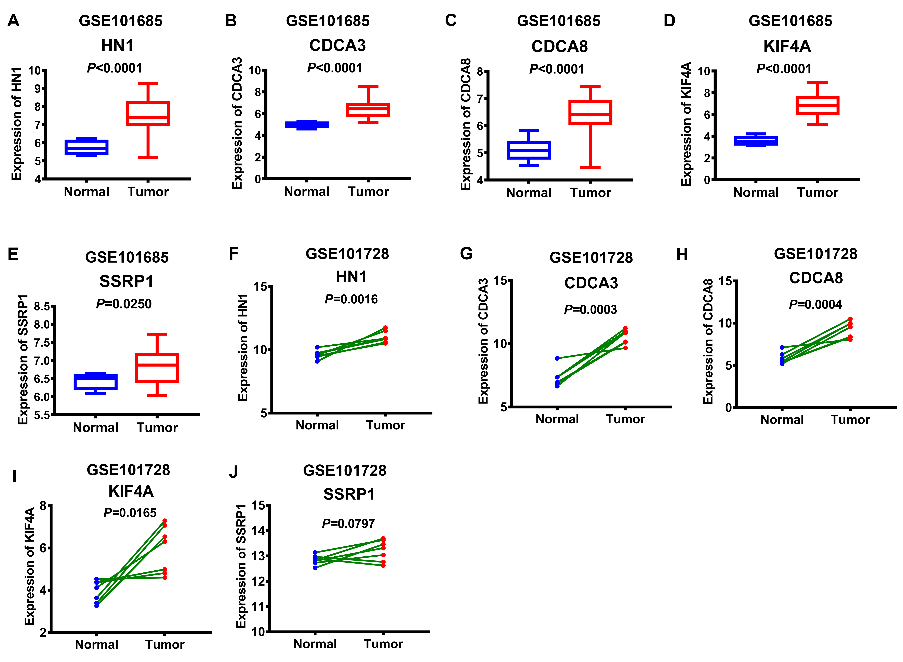


**Supplementary Figure 4 |** The expression of E2F target genes in tumor tissues versus normal tissues from GEO, using unpaired and paired t tests. **(A-E)** Unpaired t test, GSE101685, the expression of HN1, CDCA3, CDCA8, KIF4A and SSRP1 in tumor tissues versus normal tissues, respectively. **(F-J)** Paired t test, GSE101728, the expression of HN1, CDCA3, CDCA8, KIF4A and SSRP1 in tumor tissues versus normal tissues, respectively.


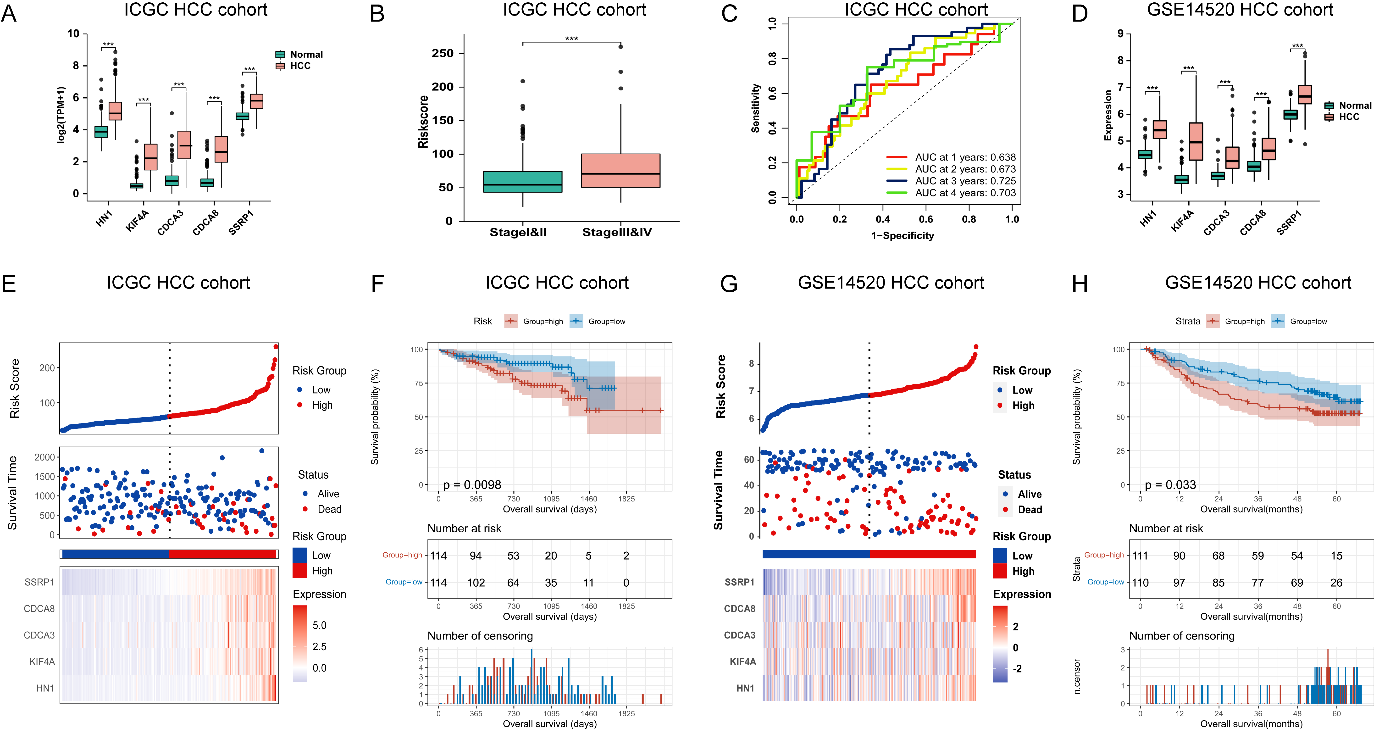


**Supplementary Figure 5 |** The external validation sets ICGC and GSE14520. **(A)** The expression levels of the 5-gene in tumor versus normal tissues in ICGC database. **(B)** The riskscore of patients in different stages of ICGC database. **(C)** ROC curve of the signature for 1-, 2-, 3- and 5-year overall survival. **(D)** The expression levels of the 5-gene in tumor versus normal tissues in GSE14520. **(E)** The risk score distribution, survival status, survival time and expression profile of the 5-gene signature of ICGC database. **(F)** The Kaplan–Meier survival analysis of the signature of ICGC database. **(G)** The risk scores distribution, survival status, survival time and expression profile of the 5-gene signature of GSE14520 set. **(H)** The Kaplan–Meier survival analysis of the signature of GSE14520 set. **p* < 0.05, ***p* < 0.01, ****p* < 0.001

**
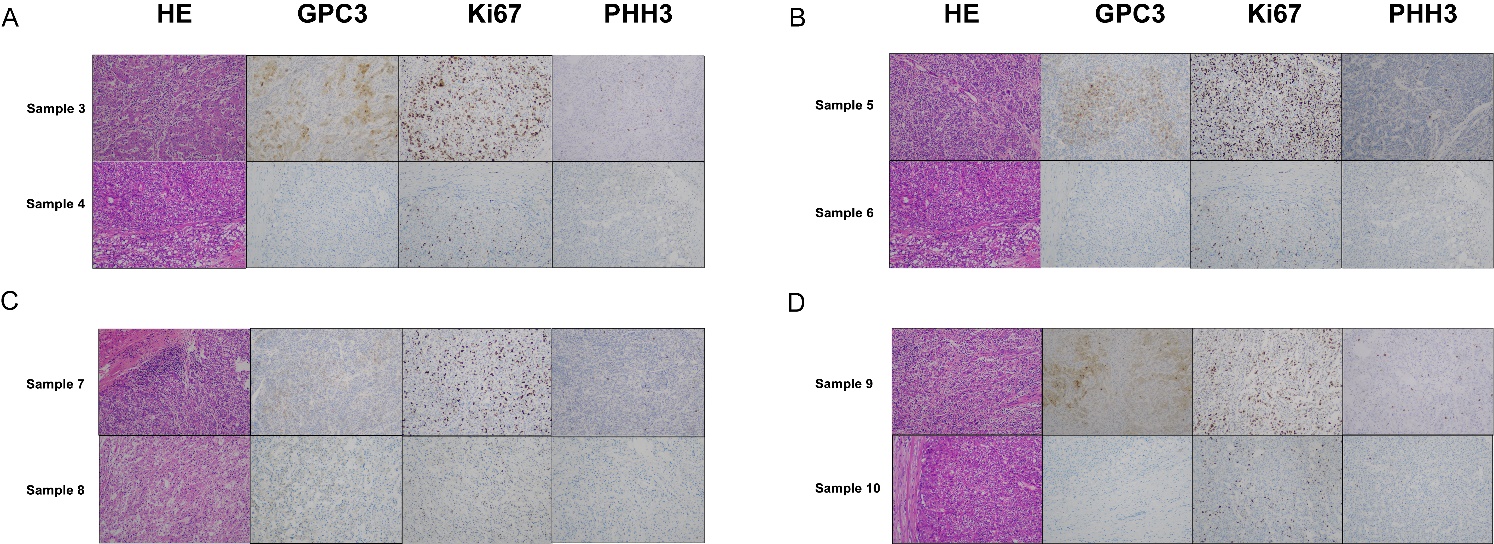
**

**Supplementary Figure 6 |** Validation of the panel signature in HCC patients from more clinical tissue specimens. **(A-D)** Representative hematoxylin-eosin (H&E) and immunohistochemistry (IHC) staining of GPC3, Ki67 and PHH3 in HCC patients.
